# Domain Adaptation for Pedestrian Detection Based on Prediction Consistency

**DOI:** 10.1155/2014/280382

**Published:** 2014-06-10

**Authors:** Yu Li-ping, Tang Huan-ling, An Zhi-yong

**Affiliations:** ^1^Key Laboratory of Intelligent Information Processing, Universities of Shandong (Shandong Institute of Business and Technology), Yantai 264005, China; ^2^School of Computer Science and Technology, Shandong Institute of Business and Technology, Yantai 264005, China

## Abstract

Pedestrian detection is an active area of research in computer vision. It remains a quite challenging problem in many applications where many factors cause a mismatch between source dataset used to train the pedestrian detector and samples in the target scene. In this paper, we propose a novel domain adaptation model for merging plentiful source domain samples with scared target domain samples to create a scene-specific pedestrian detector that performs as well as rich target domain simples are present. Our approach combines the boosting-based learning algorithm with an entropy-based transferability, which is derived from the prediction consistency with the source classifications, to selectively choose the samples showing positive transferability in source domains to the target domain. Experimental results show that our approach can improve the detection rate, especially with the insufficient labeled data in target scene.

## 1. Introduction

Recently, pedestrian detection has received much attention in the computer vision community. An accurate and fast pedestrian detector is especially useful for various vision applications such as surveillance [1, 2], intelligent vehicles [3, 4], and victim detections [5]. The general method for detecting pedestrian is a window-scanning approach, where rectangular windows of variable scales scanning densely on the image are classified as pedestrian or nonpedestrian. The great progress has been made in this field. Dalal and Triggs [6] proposed the grids of histograms of oriented gradient (HOG) descriptors with a linear SVM classifier and showed experimentally that their feature set significantly outperformed existing features sets for human detection. The powerful deformable part models (DPMs) [7] have shown promising results on challenging datasets. In this model, the appearance of each part and the deformation among parts were considered, respectively, and the star model was used to model the deformation. However, it is still a challenge to train a pedestrian detector working reliably because of wide variability in shape and appearance of the human figure. The problem is further compounded as illumination conditions, resolutions, and viewpoints change. One way to solve this problem is to require an enormous training set to handle the large uncertainty of the human's visual appearance. However, it is often impractical to collect such a large training set. Moreover, a large number of experiments show that a pedestrian detector trained using a general dataset may suffer a large performance drop in a specific target scene. For example, a general pedestrian detector trained from INRIA dataset is evaluated on the Caltech pedestrian dataset, and the absolute performance is quite unsatisfied with a log-average miss rate of over 80% [4]. This is due to the mismatch between the training INRIA set and the testing Caltech pedestrian dataset on illumination, resolutions, and viewpoint. In fact, the variation is relatively smaller in a specific scene especially in surveillance scene, since videos captured from fixed cameras only have limited variations on viewports, resolutions, and backgrounds. Therefore, it is easier to train a scene-specific pedestrian detector with high quality using samples captured from the target scene. The obvious solution would be to retain data from the relevant target scene; however, this often leads to overfitting because the target data are expensive to label and thus their quantities are limited. How to train an accurate detector using a handful of data from generic scenes and scared target data is a critical problem.

Techniques for addressing learning problems with mismatched distributions are often referred to as domain adaptation or transfer learning. In this area, the source domains refer to the labeled samples from generic scenes, while the target domain refers to the data from target scene. In addition, it is always assumed that the source and target tasks are the same, while the source and target domains are different. In general, data from source and target domains are semantically related to each other in the sense. That is, the certain parts of data in the source domains can still be reused as addition training data for learning the target model. This learning technology is especially referred to as instance transfer [8].

Approaches to domain adaptation can be divided into two cases: one case is from only one source domain transferring to the target domain; another case is from multiple source domains to the target domain. In this paper, we try to investigate the latter case. For the former, the intuitive idea is to weight a source sample based on the similarity between it and the *K* nearest target samples directly. However, a source domain is usually a mixture of several components. In this case, the distribution difference among multiple components is not exploited, but this information is very important to derive the common nature of the multiple source components.

In this paper, we propose a novel boosting-based domain adaptation method to select data from multiple source domains and learn the target model for pedestrian detection. TrAdaBoost [9] is the first algorithm of boosting to domain adaptation, and then TransferBoost [10] is proposed as an improvement over it. TransferBoost algorithm introduces a task-based notion, namely, transferability, into standard Adaboost to weight the source samples, and the transferability of a source domain is defined as the difference in weighted errors on the target domain between learning with and without this source domain transfer. However, in this paper the distribution difference among multiple source domains is stressed, and then an instance-based transferability is given. Distributions of source domains are different but related to each other; we may discover the common nature from multiple source domains. This common nature belongs to all source domains and is helpful for learning the target model. In our model, given *m* source domains, we train *m* source classifiers, respectively, on each of these source domains. Then each source instance is reweighted and transferred into the target domain according to the degree of consistency in these predictions by multiple source classifiers. That is, the source instant will be granted the maximum weight if all the source classifiers reach a perfect agreement on these prediction results. Similarly, the source instant will be granted the minimum weight if all the source classifiers get opposite opinions.

Finally, following a careful theoretical analysis, we validate the proposed method in the context of scene-specific pedestrian detection. The experimental results show that our approach can improve the detection rate, especially with the insufficient labeled data in target scene.

*Organization of the Paper.* The following sections are organized as follows. In Section 2, we review the previous work on domain adaptation and scene-specific pedestrian detection. In Section 3, we present our method and parameters definition in detail. We report experimental results in Section 4. And, finally, some brief conclusions are given in Section 5.

## 2. Related Work

Domain adaptation has received much attention in recent years. There is an extensive literature on this subject. Except for the above TrAdaBoost and TransferBoost algorithm, Cao et al. [11] studied the domain adaptation problem for face verification, combining a KL-divergence based prior knowledge with a likelihood function. Shi and Sha [12] defined an information-theoretic metric which balanced two forces: maximizing domain similarity and minimizing the expected classification error on the target domain. Chen et al. [13] combined cotraining learning with domain adaptation and formulated a single optimization problem which simultaneously learned a target predictor and a subset of source and target features.

Another closely related task is detecting scene-specific pedestrians, and existing research on this area is limited compared with extensive literature on generic pedestrian detections. Wang et al. [2] proposed a transfer learning framework to adapt a generic pedestrian detector to a specific scene. They weighted the source samples by exploring the indegrees from target samples to source samples on graph. Moreover, they took the context cues from motions, scene structures, and scene geometry to select samples from the target scene. However, motion based detection is not robust due to many factors, such as shadows and illumination change. Wu and Nevatia [14] presented an unsupervised, online retaining approach based on online boosting, and the oracle (labeler) for unsupervised learning was based on combination of a set of part detectors learned by offline boosting. The main issue of unsupervised learning is a labeler's errors and the selected target samples are likely to have wrong label or inaccurate position or size which may make the detector drift.

## 3. Domain Adaptation Algorithm

In this section, we first define the problem of domain adaptation from multiple source domains to the target domain, and then the framework of domain adaptation algorithm is proposed. Finally, how to choose parameters is introduced.

### 3.1. Problem Definition

Let *S*_1_,…*S*_*m*_ be *m* (*m* > 1) source domains with labeled instances, and the *i*th source dataset is represented as *S*_*i*_ = {(*x*_*j*_, *y*_*j*_)}_*j*=1_^|*S*_*i*_|^, where *y*_*j*_ = ±1 is the label of instance *x*_*j*_. These data from source domains are assumed to be abundant. The target domain is denoted by *T* = {(*x*_*j*_, *y*_*j*_)}_*j*=1_^*n*^, where *n* is the number of instances in the target domain. The quantity of the target data is often insufficient to learn a good model for the target domain. Under the assumption that the distributions of *S*_1_,…, *S*_*m*_ and *T* are different but related to each other, the goal of domain adaptation is to train a classifier *f* for the target domain, selecting instances from *S*_1_,…, *S*_*m*_ into the target dataset *T* in learning the model.

### 3.2. Domain Adaptation Algorithm

Our proposed domain adaptation model is summarized in Algorithm 1, which is based on TransferBoost [11] algorithm. First, data from multiple source domains and the target domain are merged into the whole training dataset *D*. Then, instances from both multiple source domains and target domain are reweighted iteratively in different methods. On each iteration *t*, given instances from training dataset *D* with a distribution *w*_*t*_(*D*), the base learning algorithm computes a weak hypothesis *h*_*t*_; then, for the purpose of minimizing the weighted error *ε*_*t*_, *α*_*t*_ is computed and the details are provided in Section 3.4. Moreover, source classifier *H*_*t*_^*i*^ *i* = 1,…, *m* is trained individually on source domain *S*_*i*_ with distribution *w*_*t*_(*S*_*i*_) according to the standard Adaboost paradigm. For each source instance, *β*_*t*_(*x*_*j*_) is computed according to the transferability to the target domain and detailed in Section 3.3. Furthermore, the instance is reweighted in this model as follows. The weight of instance in the target domain *T* is increased due to whether this instance is mispredicted by weak hypothesis *h*_*t*_ following the standard Adaboost algorithm; however, each instance in the source domains increases or decreases its weight by two terms in (∗) of Algorithm 1. The first term ensures the mispredicted instances with higher weight in a similar way, and then the second term *β*_*t*_(*x*_*j*_) denotes the transferability from the source instance *x*_*j*_ to the target domain *T*. Our intuition in this model is that the source instances with a high agreement predicted by all the source classifiers could yield better transferability across domains. The coefficient *λ* controls the balance between these two terms. After several iterations, the final classifier is constructed by a weighted sum of the weak classifiers.

### 3.3. Choosing the *β*_*t*_(*x*_*j*_) Parameter

In instance transfer, the instance which shows positive transferability should be transferred into the target domain with higher weight, whereas the instance with negative transferability should avoid to be transferred. Thus, our challenge is how to define the transferability from a source instance to the target domain.

To address this challenge, we first describe how to measure the consistent degree of prediction by multiple source classifiers. From an information-theoretic perspective, the Shannon entropy, which quantifies the uncertainty in a random variable, may be used as a useful candidate metric for consistency. Given a set of classifiers {*H* ^*i*^}_*i*=1_^*m*^, each of which may assign confidence score to an instance *x*_*j*_, we get a probability distribution vector *P* ^*i*^(*x*_*j*_). The average probability distribution vector may be calculated as

(1) $$\begin{array}{c} \overset{-}{P}\left(x_{j}\right)=\frac{\sum_{i=1}^{m}P^{i}\left(x_{j}\right)}{m}. \end{array}$$

Moreover, based on the Shannon entropy, a definition to measure the consistent degree of prediction by multiple classifiers is given by

(2) $$\begin{array}{c} C\left(P^{1}\left(x_{j}\right),\ldots ,P^{m}\left(x_{j}\right)\right)=-\;E\left(\overset{-}{P}\left(x_{j}\right)\right), \end{array}$$

where *E*(·) denotes the Shannon entropy and $\overset{-}{P}(x_{j})$ is the average probability distribution vector defined in (1).

Intuitionally, the source instant *x*_*j*_ will show positive transferability if all the source classifiers reach a high agreement on these prediction results. Similarly, the source instant *x*_*j*_ will show negative transferability if all the source classifiers get opposite opinions. Consequently, transferability from a source instance *x*_*j*_ to the target domain in iteration *t* is described using parameter *β* ^*t*^(*x*_*j*_) as

(3) βt(xj)=C(P1(xj)+⋯+Pm(xj))=− E(P¯(xj)).

### 3.4. Choosing the *α*_*t*_ Parameter

Following TransferBoost algorithm, the following bound holds on the training error *ε*_*T*_ = (1/|*T*|)∑_∀*x*_*j*_∈*T*_ *ε*_*j*_:

(4) $$\begin{array}{c} \varepsilon_{T}\leq \frac{\left|D\right|}{\left|T\right|}\overset{K}{\underset{t=1}{\prod }}Z_{t}, \end{array}$$

where *ε*_*j*_ = ⟦*H*(*x*_*j*_) ≠ *y*_*j*_⟧  ((*x*_*j*_, *y*_*j*_) ∈ *T*) is the error of the instance (*x*_*j*_, *y*_*j*_) suffered by the classifier *H*, *K* is the maximum number of iterations, and *Z*_*t*_ is a normalization factor. To minimize the bound, we choose the following parameter:

(5) $$\begin{array}{c} \alpha_{t}=\frac{1}{2}\ln\left(\frac{1+\sum_{x_{j}\in D}w_{t}\left(x_{j}\right)y_{j}h_{t}\left(x_{j}\right)}{1-\sum_{x_{j}\in D}w_{t}\left(x_{j}\right)y_{j}h_{t}\left(x_{j}\right)}\right). \end{array}$$

## 4. Experiments

**Figure 1:**
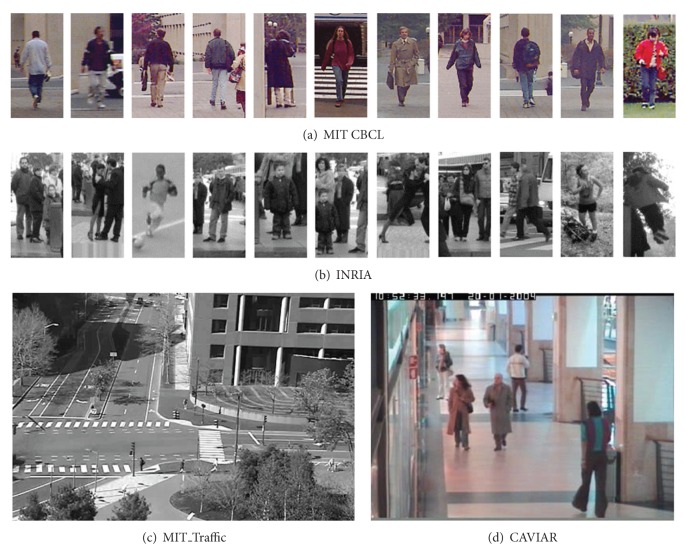
Some samples and example images (cropped) from four datasets.

For evaluating our domain adaptation model, we employed two generic-scene datasets (MIT CBCL [15] and INRIA [16]) and two specific-scene datasets (MIT_Traffic [17] and CAVIAR [18]), respectively. Some pedestrian samples and example images are shown in Figure 1. Decision stump is used as base learning algorithm and HOG descriptor is adopted as the feature representation for the whole test. The false positive per image (FPPI) versus recall rate is used as the evaluation metric.

### 4.1. Datasets

The one generic-scene dataset is obtained from the MIT CBCL dataset, where the pedestrian instances are less varied in pose and illumination and in front and rear viewpoint. 924 pedestrian samples (1848 with their left-right reflections) are exacted for training. Another more comprehensive dataset is INRIA dataset. The INRIA pedestrian dataset consists of the training set and the testing set. In our implements, only 1208 pedestrian samples (2416 with reflections) in training set are used. The pedestrians in the INRIA dataset are in various poses, illumination, and viewpoints, and some of them are partially occluded.

The two specific-scenes surveillance datasets, MIT_Traffic and CAVIAR, are also involved in our tests. MIT_Traffic dataset is captured at 30 fps from the real street intersection. These video sequences contain more challenges due to perspective deformation. For the training set, 210 frames with 790 pedestrian instances are uniformly sampled from the first part of the video sequences; meanwhile, 100 frames are uniformly exacted from the rest of video sequences for testing. CAVIAR dataset is captured by a stationary camera with a wide angle lens. 118 frames with 569 pedestrian instances are uniformly sampled from WalkbyShop1cor clip for training, and then 165 frames are uniformly sampled from ShopAssistant2cor clip for testing.

Table 1 shows the descriptions for training sets in our experiments. The first two datasets are used to train our domain adaptation model, respectively, in different target domain. The name of the dataset MIT_Traffic_DA indicates that MIT_Traffic dataset is the target domain in domain adaptation, and the other three datasets are used as source domains. The dataset, CAVIAR_DA, is named in the same way. Furthermore, we also constructed the next two datasets with samples only from the target domain. Finally, the last dataset consists of all 5623 training samples. In the experiments, we also randomly sampled 6000 negative samples from the INRIA's training negative images (10 for each image) as the training negative set. The descriptions for testing set are shown in Table 2. The dataset for testing is named according to the target scene.

### 4.2. Experiments Results

**Figure 2:**
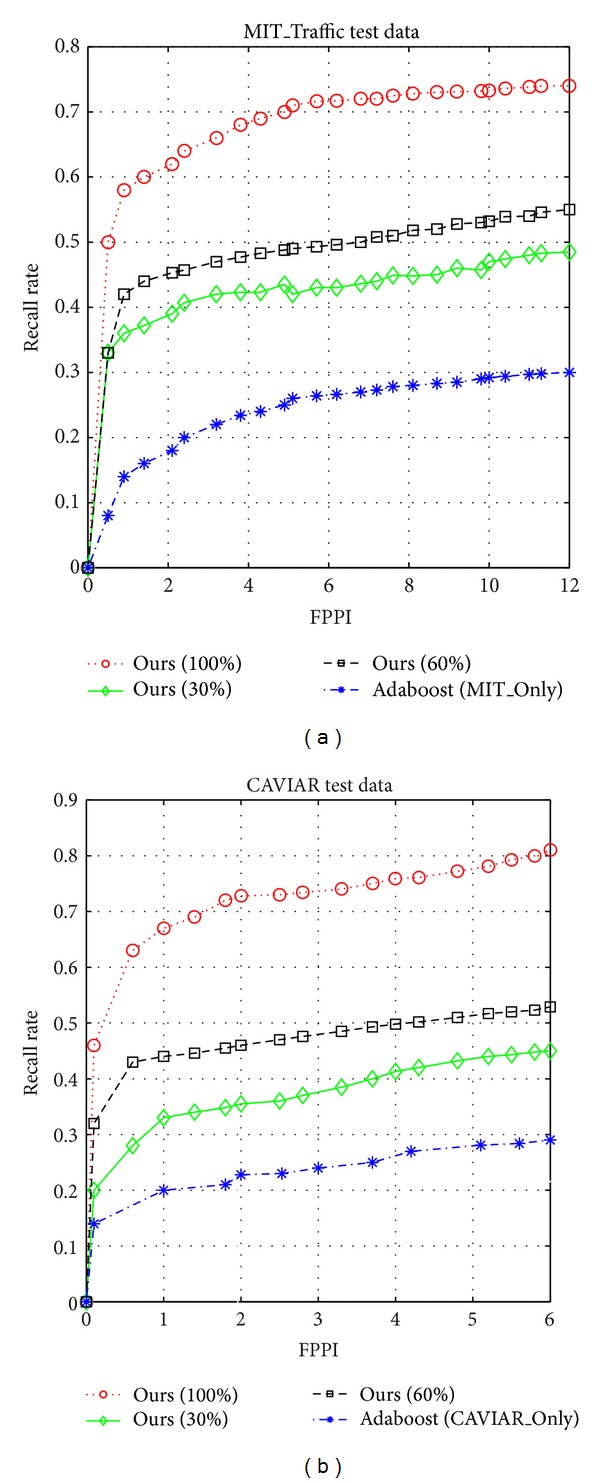
Results on MIT_Traffic scene (a) and CAVIAR scene (b). (a) and (b) compare with Adaboost algorithm trained only on the target domain dataset and our approach trained on different numbers of the source data.

In the experiments, we first evaluated the proposed domain adaptation model when trained on 30%, 60%, and 100% of the source training data. The algorithm *Adaboost*(*T*), trained on only the target domain *T*, was also involved in this experiment.  Figure 2 shows their learning curves on two target scenes, respectively. In the following discussion, when the recall rate is mentioned, we always assume that FPPI = 2. In Figure 2, the results indicate that the recall rate of our model increases with the increase of the number of source data. Particularly, the proposed model significantly outperforms the Adaboost algorithm trained on only the target domain. On the MIT_Traffic test dataset, detection rate is improved from 18% to 62%, and, on the CAVIAR test dataset, detection rate is improved from 23% to 73%. This is because the quantity of target data is too small to train a high-efficient pedestrian detector. Experiments also show that the proposed approach performs significantly worse on MIT_Traffic scene. The reason for that may lie in MIT_Traffic dataset with more perspective deformation and lower resolution compared with CAVIAR dataset.

**Figure 3:**
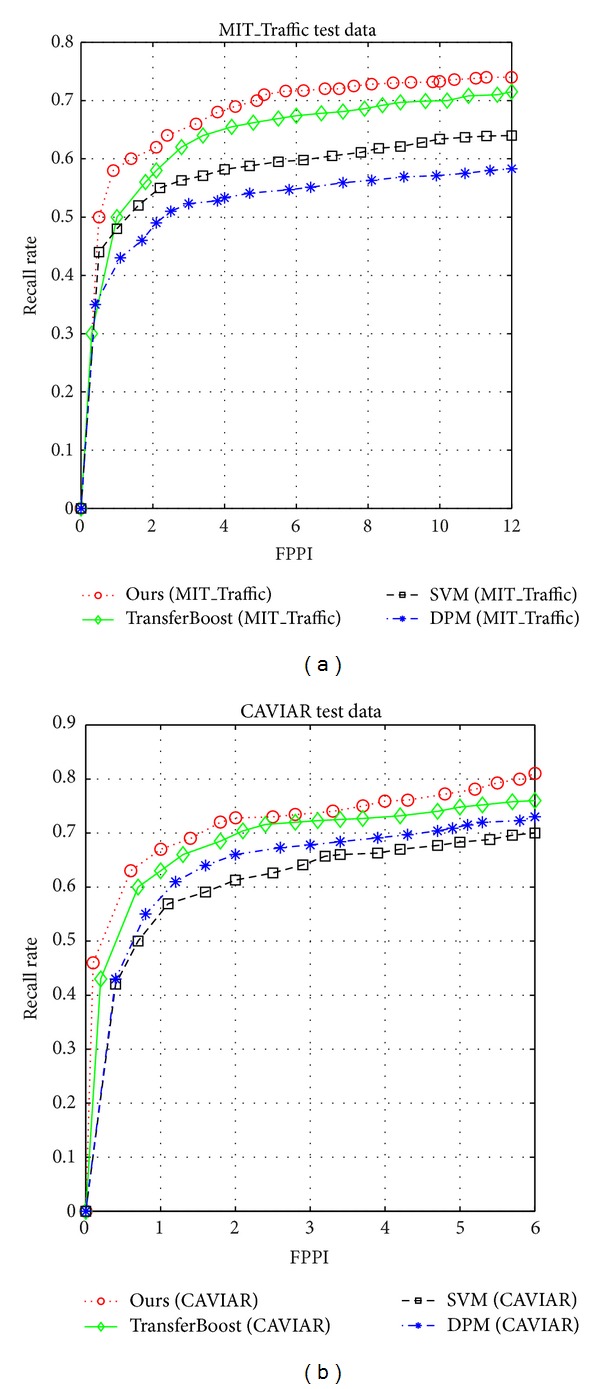
Comparison with TransferBoost, linear SVM, and DPM on MIT_Traffic scene (a) and CAVIAR (b) scene.

Furthermore, Figure 3 compares three algorithms: TransferBoost [10] algorithm, linear SVM [6], and DPM [7] in two target scenes, respectively.  Table 3 describes the learning methods in detail. This graph shows that our approach, with the higher recall rate, exhibits competitive results compared with the other three algorithms. On the MIT_Traffic test dataset, compared with TransferBoost, near 7% gain is achieved by our approach, and, on the CAVIAR test dataset, the gain is about 4%. In addition, it outperforms the generic detectors, SVM detector, and DPM detector, trained on both source and target data. It may be due to the fact that proper using of the source data and avoiding negative transferability can improve the generalization power.

## 5. Conclusions and Future Work

In this paper, we proposed a novel domain adaptation model for pedestrian detection in a specific scene especially in video surveillance. The main idea is to transfer the source instances to a target domain using the common nature among multiple source domains. Accordingly, each source instance is reweighted and transferred into the target domain according to the degree of consistency in the predictions by multiple source classifiers. Moreover, the effectiveness of the proposed algorithm is evaluated through experiments on two video surveillance datasets.

We note that a large number of source instances should be stored when transferring to a specific scene in our approach, and it is inefficient in practical applications. In the future, we will try to extend the model to parameterize the knowledge of multiple source domains.

## Figures and Tables

**Algorithm 1:**
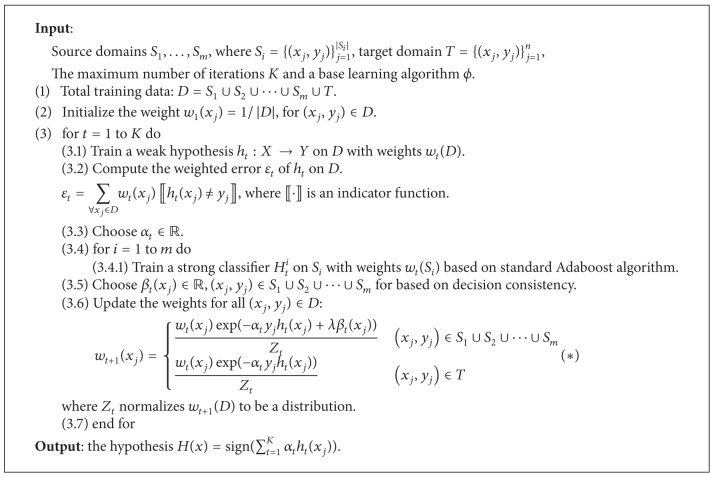
The proposed domain adaptation algorithm.

**Table 1:** The descriptions of the datasets for training.

Dataset	Training set	
	Positives	Negatives
MIT_Traffic_DA	Source domains: total of 4833 samples, INRIA, MIT CBCL, and CAVIAR WalkbyShop1cor (118 frames) Target domain: 790 samples, MIT_Traffic (top 210 frames)	6000 samples and INRIA
CAVIAR_DA	Source domains: total of 5054 samples, INRIA, MIT CBCL, and MIT_Traffic (top 210 frames) Target domain: 569 samples and CAVIAR WalkbyShop1cor (118 frames)	
MIT_Only	790 samples and MIT_Traffic (top 210 frames)	
CAVIAR_Only	569 samples and CAVIAR WalkbyShop1cor (118 frames)	
All	Total of 5623 samples and INRIA + MIT CBCL + MIT_Traffic (top 210 frames) + CAVIAR WalkbyShop1cor (118 frames)	

**Table 2:** The descriptions of the datasets for testing.

Target scene	Test set
MIT_Traffic	MIT_Traffic (bottom 100 frames)
CAVIAR	CAVIAR ShopAssistant2cor (165 frames)

**Table 3:** The descriptions of learning methods.

Learning method	Training data	Testing data
Ours (MIT_Traffic)	MIT_Traffic_DA	MIT_Traffic
Ours (CAVIAR)	CAVIAR_DA	CAVIAR
TransferBoost (MIT_Traffic)	MIT_Traffic_DA	MIT_Traffic
TransferBoost (CAVIAR)	CAVIAR_DA	CAVIAR
SVM (MIT_Traffic)	All	MIT_Traffic
SVM (CAVIAR)	All	CAVIAR
DPM (MIT_Traffic)	All	MIT_Traffic
DPM (CAVIAR)	All	CAVIAR

## References

[1] Aggarwal JK, Ryoo MS (2011). Human activity analysis: a review. *ACM Computing Surveys*.

[2] Wang M, Li W, Wang X Transferring a generic pedestrian detector towards specific scenes.

[3] Gerónimo D, López AM, Sappa AD, Graf T (2010). Survey of pedestrian detection for advanced driver assistance systems. *IEEE Transactions on Pattern Analysis and Machine Intelligence*.

[4] Dollár P, Wojek C, Schiele B, Perona P (2012). Pedestrian detection: an evaluation of the state of the art. *IEEE Transactions on Pattern Analysis and Machine Intelligence*.

[5] Andriluka M, Schnitzspan P, Meyer J Vision based victim detection from unmanned aerial vehicles.

[6] Dalal N, Triggs B Histograms of oriented gradients for human detection.

[7] Felzenszwalb PF, Girshick RB, McAllester D, Ramanan D (2010). Object detection with discriminatively trained part-based models. *IEEE Transactions on Pattern Analysis and Machine Intelligence*.

[8] Pan SJ, Yang Q (2010). A survey on transfer learning. *IEEE Transactions on Knowledge and Data Engineering*.

[9] Dai W, Yang Q, Xue G-R, Yu Y Boosting for transfer learning.

[10] Eaton E, Desjardins M Selective transfer between learning tasks using task-based boosting.

[11] Cao X, Wipf D, Wen F, Duan G A practical transfer learning algorithm for face verification.

[12] Shi Y, Sha F Information-theoretical learning of discriminative clusters for unsupervised domain adaptation.

[13] Chen M, Weinberger KQ, Blitzer JC Co-training for domain adaptation.

[14] Wu B, Nevatia R Improving part based object detection by unsupervised, online boosting.

[15] http://cbcl.mit.edu/software-datasets/PedestrianData.html.

[16] http://pascal.inrialpes.fr/data/human/.

[17] Wang X, Ma X, Grimson WE (2009). Unsupervised activity perception in crowded and complicated scenes using hierarchical bayesian models. *IEEE Transactions on Pattern Analysis and Machine Intelligence*.

[18] http://homepages.inf.ed.ac.uk/rbf/CAVIARDATA1/.

